# Evolutionary Diversity and Function of Metacaspases in Plants: Similar to but Not Caspases

**DOI:** 10.3390/ijms23094588

**Published:** 2022-04-21

**Authors:** Sung Un Huh

**Affiliations:** Department of Biological Science, Kunsan National University, Gunsan 54150, Korea; sungun@kunsan.ac.kr

**Keywords:** caspase, metacaspase, programmed cell death, protease, autophagy

## Abstract

Caspase is a well-studied metazoan protease involved in programmed cell death and immunity in animals. Obviously, homologues of caspases with evolutionarily similar sequences and functions should exist in plants, and yet, they do not exist in plants. Plants contain structural homologues of caspases called metacaspases, which differ from animal caspases in a rather distinct way. Metacaspases, a family of cysteine proteases, play critical roles in programmed cell death during plant development and defense responses. Plant metacaspases are further subdivided into types I, II, and III. In the type I Arabidopsis MCs, AtMC1 and AtMC2 have similar structures, but antagonistically regulate hypersensitive response cell death upon immune receptor activation. This regulatory action is similar to caspase-1 inhibition by caspase-12 in animals. However, so far very little is known about the biological function of the other plant metacaspases. From the increased availability of genomic data, the number of metacaspases in the genomes of various plant species varies from 1 in green algae to 15 in Glycine max. It is implied that the functions of plant metacaspases will vary due to these diverse evolutions. This review is presented to comparatively analyze the evolution and function of plant metacaspases compared to caspases.

## 1. Basic Features of Caspase and Metacaspase

**Figure 1:**
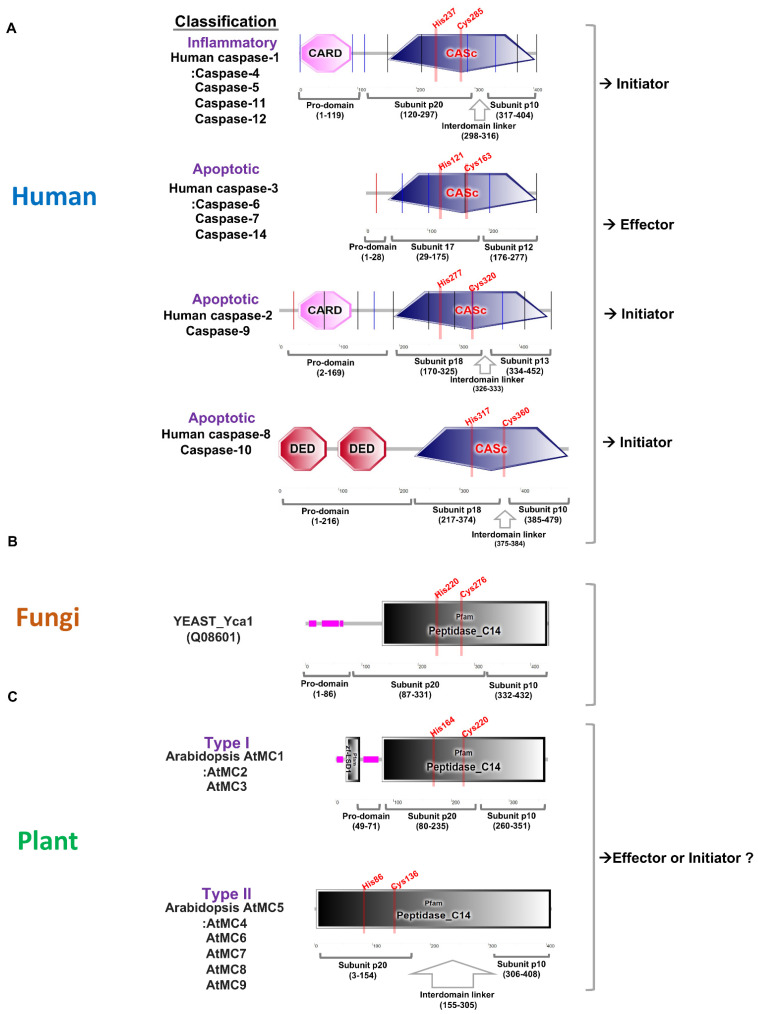
Comparative analysis of protein domains for caspase and metacaspase in human, yeast, and plant. (**A**). Protein domains for human caspase-1, caspase-2, and caspase-3 are marked using a simple modular architecture research tool (SMART) [14]. Caspases are classified into inflammatory and apoptotic according to their functions, and apoptotic caspases are further divided into initiators and effectors. Initiator caspases have a pro-domain containing a CARD, or death effector domain (DED), at the N-terminus, and effector caspases have a very short pro-domain. Human caspases have conserved active sites (His and Cys) in the large subunit p20. (**B**). Yeast contains one metacaspase in its genome and has a pro-domain in the N-terminus. There is no special linker between subunits p20 and p10, and active sites (His and Cys) are conserved in subunit p20. (**C**). *Arabidopsis thaliana* contains three type I and six type II metacaspases, and type I has an N-terminal pro-domain with or without a zinc-finger at the N-terminus. Type II lacks a pro-domain, but it is characterized by a long linker between subunits p20 and p10. The active sites (His and Cys) are conserved in subunit p20.

Apoptosis is one of the programmed cell deaths that is crucial for tissue development and homeostasis [1]. Programmed cell death is promoted by caspases, a highly conserved set of intracellular proteases. Caspase is a term used to describe the two functional roles of this group of enzymes. In this case, “c” stands for cysteine protease, and “aspase” stands for its ability to cleave aspartic acid residues [2]. Based on caspase functions, mammalian caspases are divided into the apoptotic and inflammatory caspases groups. Inflammatory caspases trigger a form of inflammation known as pyroptosis. These caspases play important roles in the activation of the inflammasome to initiate inflammation and initiate programmed cell death. Inflammatory caspases contain caspase-1, caspase-4, caspase-5, caspase-11, and caspase-12 (Figure 1A). On the other hand, apoptotic caspases initiate and execute an immunologically silent form of programmed cell death known as apoptosis. Apoptotic caspases are subgrouped into initiator and effector caspases according to their functional order in the execution of apoptosis [3]. Initiator caspases, including caspase-2, caspase-8, caspase-9, and caspase-10, function as proteolytic signal amplifiers to activate effector caspases. Effector caspases, including caspase-3, caspase-6, and caspase-7 enhance apoptosis through the proteolysis of several cellular proteins at their target sites (Figure 1A).

Caspases are comprised of a pro-domain and a mature chain, which is folded into a caspase/hemoglobinase. After the removal of the pro-domain, caspases undergo proteolytic cleavage that releases two peptide fragments with the large subunit p20 (about 20 kDa) and small subunit p10 (about 10 kDa), respectively. Caspase-1, caspase-2, caspase-4, caspase-5, caspase-9, caspase-11, and caspase-12 also have amino-terminal pro-domains including caspase recruitment domains, or caspase activation and recruitment domains (CARDs), which are interaction motifs. Effector caspase-3, caspase-6, and caspase-7 contain short pro-domains and these caspases are activated by the initiator caspases (Figure 1A). Caspase-1 can, in basic terms, induce pyroptotic cell death in response to pathogen- associated signals, and is, therefore, critical for innate immunity [4]. The full-length pro-caspase-1 is activated by proximity-induced autologous proteolysis. Activated caspase-1 cleaves the inflammatory cytokines pro-IL-1β and pro-IL-18 for activating cytokines [5]. Caspase-1, including caspase-4, -5, and -11, can specifically cleave pore-forming protein gasdermin D (GSDMD), and cleavage of GSDMD is required for pyroptosis [5,6]. Interestingly, interdomain linker cleavage of caspase-1 is also required for pyroptosis [7]. Although caspase cleavage occurs in various domains, it can be expected that the activation step is highly conserved in the caspases of other species. Many metacaspases were not found in fungi compared to plants. The yeast genome contains only one metacaspase, called Yca1, which has a long pro-domain and undergoes autocatalytic processing in a Ca^2+^-dependent manner [8]. Yca1 has a protein fold similar to the canonical caspases (Figure 1B). Yca1 is known to play an important role as a positive regulator of apoptosis [9,10,11]. The protease activity of Yca1 is believed to degrade protein aggregates rather than limit aggregate formation [12]. The rice blast fungus *Magnaporthe oryzae* has two metacaspase genes, *MoMca1* and *MoMca2* [13]. It has been reported that these metacaspase proteins exhibit functional redundancy and can complement the yeast Yca1 mutant [13]. Double mutant *Momca1mca2* strain contains increased insoluble aggregates during vegetative growth. MoMca1 and MoMca2 promote the clearance of the insoluble aggregates in *M. oryzae* [13]. Metacaspase function in fungi may help maintain the fitness of fungal cells by eliminating insoluble aggregates under stress conditions.

The caspase family is the only family of cysteine proteases with members in all kingdoms according to the MEROPS database [15,16]. Plant genomes also contain evolutionarily conserved caspase-like genes. However, plant proteases are not cysteine-dependent aspartate-directed proteases, which are characterized as mammalian caspases, but structural homologues called metacaspases [17]. In a previous report, phylogenetic analysis found that eukaryotic caspases, metacaspases, and paracaspases were equally distant from each other. It is classified within the clade CD of cysteine protease [17]. Metacaspases are only found in eukaryotes such as plants, fungi, and protists. As in caspases, metacaspases contain a caspase-specific catalytic dyad of histidine and cysteine in the large subunit p20. Although plant metacaspases lack substrate specificity for aspartate residues, metacaspases share structural homology with mammalian caspases. Based on the presence or absence of an N-terminal pro-domain, three types of metacaspases, containing subunit p20 and subunit p10 caspase domains, are identified [15,18]. Type I metacaspases contain an N-terminal pro-domain containing a proline-rich repeat motif and a zinc finger motif in plant members (Figure 1C). There are nine metacaspases in Arabidopsis, of which AtMC1, AtMC2, and AtMC3 have the characteristics of type I metacaspases. Type II metacaspases lack a pro-domain at the N-terminus but present a linker region between the putative large subunit p20 and small subunit p10. AtMC4-AtMC9 have the characteristics of type II (Figure 1C). The AtMC4 crystal structure is determined and AtMC4 can modulate Ca2^+^-dependent as a damage-induced plant immune defense [19]. Large linker domain of AtMC4 acts as inhibitory conformation and suppresses metacaspase activation [19]. Type II metacaspases appear to have evolved functions to regulate protein activity through a long linker domain between p20 and p10 instead of at the N-terminal extension. Recently, type III metacaspases were discovered in the genome of the *Guillardia theta* algae. [20]. *G. theta* metacaspases exhibit 1 type I (GtMC1), 1 type III (GtMC2), and 11 metacaspase-like types (GtMC3-GtMC14) [20]. Type III differs from type I in that the protein position of p20–p10 is switched only in the order of p10–p20. More research is needed to determine what kind of functional effect this arrangement has on protease. Although studies on metacaspase-like types are still lacking, it can be determined that algae have evolved in a considerable number and in various forms.

## 2. Subcellular Localization of Caspase and Metacaspase

The intracellular localization of caspase and metacaspase shows various distributions [21,22]. In particular, their intracellular localization in the activated state may be important as caspase and metacaspase undergo autoproteolysis and interact with target proteins. Pro-caspase-1 is present in the cytoplasm in an inactive form and requires inflammasomes for proteolytic activation. Interestingly, pro-caspase-1, NLRP3 (NOD-, LRR- and pyrin domain-containing protein 3), and ASCs (apoptosis-associated speckle-like proteins with carboxy-terminal CARDs) translocate between the nucleus and the cytoplasm as a reaction to an inflammatory response [23,24,25]. 

Caspase-1 can target and cleave the GATA4 transcription factor, which regulates cardiac cell fate [26]. Mature caspase-1 can be expected to have various functions in the nucleus when activated. In addition, GATA4 was found to be evolutionarily conserved in a similar way to transcriptional regulators in plants and fungi [27,28,29,30,31,32]. GATA factors can bind to the 5′-WGATAR-3′ motif via a C-terminal zinc finger domain, and an N-terminal zinc finger lends supports in order to stabilize the interaction [33]. Although there is no known metacaspase-GATA protein interaction yet, it is likely that plant and fungal metacaspases bind to nuclear GATA transcription factors and participate in transcriptional regulation. Furthermore, caspase-2 contains a classical nuclear localization signal peptide, and also contains a putative mitochondrial targeting sequence [34]. Caspase-2 has been observed in the mitochondria, and it has been determined that it is essential for mitochondrial oxidative stress-induced apoptosis. *Casp*2^−/−^ primary skin fibroblasts are protected upon oxidant treatment [34]. A general fact is that caspase-2 activation occurs mainly in the cytoplasm, but its function is exerted at various subcellular locations [35]. Additionally, caspase-1 or caspase-3 have been detected in the plasma membrane to promote pyroptosis and apoptosis-induced proliferation [21,36]. It can be expected that caspases with these diverse intracellular localizations can be translocated depending on the targets.

The yeast genome only has a single type I metacaspase, Yca1, which localizes in insoluble protein aggregates via its N-terminal pro-domain and promotes aggregate clearance [37]. The loss of Yca1 results in increased retention of aggregated material within the insoluble aggregates. It has been found that Yca1 associates with components of the ubiquitin protease system (UPS), such as E3 ligase Rsp5 and ubiquitinated Yca1 is located in the juxtanuclear quality control compartment (JUNQ) which is tethered to the nucleus [38]. As in Yca1, the Arabidopsis full-length AtMC1 is found in the microsomal, in the insoluble fraction, but processed AtMC1 is located in the soluble fraction [39]. Interestingly, the catalytic dead mutant AtMC1 (C99A-C220A) protein remained mostly insoluble. Independent of the catalytic activity, it can be detected that the intra-cellular localization of AtMC1 is very similar to that of Yca1. During programmed cell semi-death of sieve elements in *Tritium aestivum*, type II metacaspase TaeMCAII has been detected in dynamic localizations [40]. The authors collected spikelet samples from 0 to 7 days post-flowering (DAF) to detect TaeMCAII localization using immunoelectron microscopy. In the first step (1 and 2 DAF), TaeMCAII was mainly located to the nucleus. In the middle stage (3, 4, and 5 DAF), it was generally distributed around the cytoplasm and nuclear fragments. In the last stage (6 and 7 DAF), which started at the last stage of sieve element developments, translocation of TaeMCAII from the cytoplasm to the cell wall was found [40]. These results implied that the intracellular localization of metacaspases was also shifted, almost similar to the movement of Ca^2+^. Sieve elements are different from typical programmed cell death, but it can be expected that the function of metacaspases during development is determined by changes in their subcellular localization that are dependent on Ca^2+^. 

Two grapevine (*Vitis rupestris* L.) metacaspases, VrMC2 and VrMC5 have been identified as type I and type II metacaspases, respectively [41]. VrMC2-GFP, which contains a putative retention-like motif (KPFI) in the C-terminal region, is localized around the nucleus area as aggregated dots, and is merged with the ER marker protein. On the other hand, VrMC5-GFP, which lacks any canonical organelle-targeting signal, is detected mainly in the cytoplasm and nucleus [41]. Thus, it is expected that type I and type II metacaspases may function differently at different subcellular locations in the cell. However, both VrMC2 and VrMC5 participate in cell death-related immunity and act as executors of hypersensitive cell death. The VrMC2 homologue from *Oryza sativa* OsMC1, which is localized exclusively in the nucleus, has putative nuclear localization signals (NLS) in the N-terminal region [22]. The type II OsMC5, OsMC5, and OsMC8 were normally found in the cytoplasm [22]. Similarly, Arabidopsis AtMC4 was predominantly localized in the cytosol, but was also detected in the nucleus [42,43]. Interestingly, type II AtMC4, AtMC5, AtMC6, and AtMC7 participate in processing of tonoplast-localized plant elicitor peptide 1 (PROPEP1), to active Pep-mediated plant immunity and these type II metacaspases might translocate in diverse cellular positions. Maize type I metacaspases, ZmMC1 and ZmMC2, exhibited partial localization with the autophagic marker ATG8a in maize protoplasts [44]. If coexpressed with intracellular nucleotide-binding, leucine-rich repeat (NLR or NB-LRR) protein, Rp1-D21 or coiled-coil (CC) domain of Rp1-D21, ZmMC1, and ZmMC2 proteins localize to the nucleocytoplasm and dot-like structure [44]. As in animal caspases, plant metacaspases can move to various subcellular locations in the cell. The dynamic intracellular localization of metacaspases can be influenced by protein interaction partners or targets within the cell. 

## 3. Diverse Metacaspase Gene Duplications and Conserved Cysteine Protease Features

**Figure 2:**
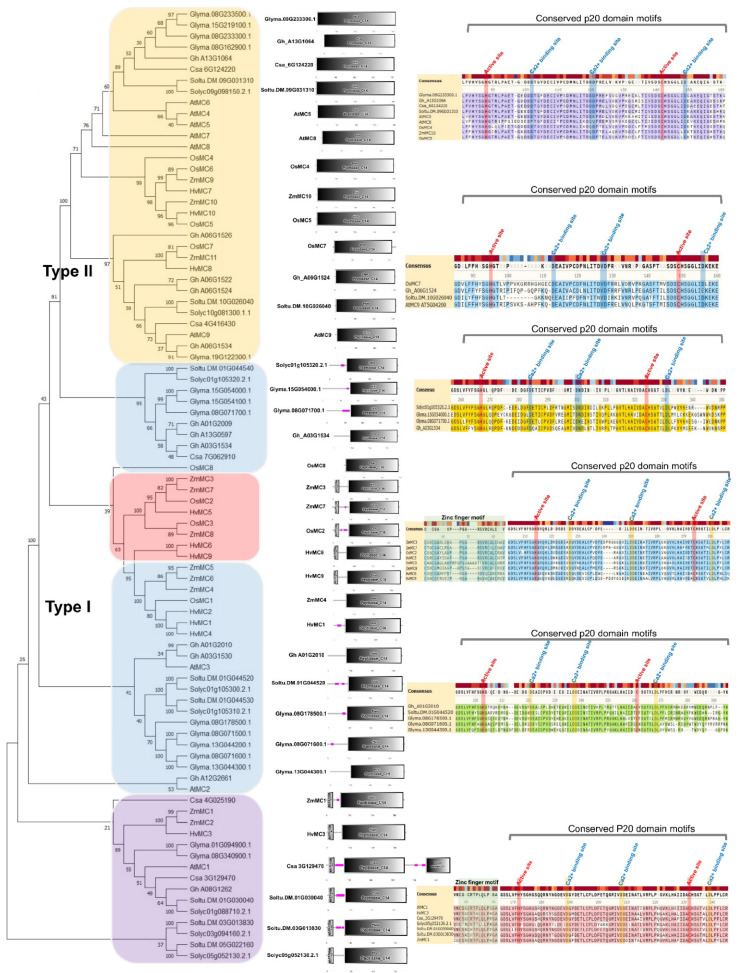
Phylogenetic tree of metacaspase gene family in plant. Multiple sequence alignment of full-length proteins was performed using ClustalW, and the phylogenetic tree was constructed using the MEGA X tool with 1000 boot strap replicates [49]. Schematic representation of the conserved domains of metacaspase proteins in *Arabidopsis thaliana* (9 AtMCs), *Hordeum vulgare* (10 HvMCs), *Brassica napus* (13 BnMCs), *Cucumis sativus* (5 CsaMCs), *Glycine max* (15 GmMCs), *Gossypium hirsutum* A genome (12 GhMCs), *Oryza sativa japonica* (8 OsMCs), *Solanum tuberosum* (8 SotubMCs), *Solanum lycopersicum* (8 SIMCs), and *Zea mays* (11 ZmMCs) using the SMART tool. Partial alignment of selected plant metacaspases in the p20 domain shows the conserved calcium (Ca^2+^) binding sites, and the active dyad (active sites). Color background represents above 50% similarity.

As genome sequencing progresses in various plant species, more and more metacaspase genes are being discovered. In general, *Arabidopsis thaliana* has a total of nine metacaspases of 3 type I and six of type II (Figure 2). Tomatoes and potatoes, the solanaceous crops, contain a total of eight metacaspases [45,46]. Unlike *Arabidopsis*, the proportion of type I metacaspases is high. It consists of six metacaspases of Type I and two metacaspases of Type II. It is also characterized by a tendency to concentrate at the same locus in the genome [45,46]. Therefore, a lot of gene duplication has occurred at the same locus [45,46]. It can be found that more metacaspase duplication occurred in important crops. Rice contains a total of 8 metacaspases, wheat contains 10, sorghum has 11, rapeseed contains 13, glycine has 15, and cotton has 12 metacaspases in the A genome (Figure 2). Interestingly, with the exception of *Arabidopsis* and cucumber, the ratio of type I metacaspases was quite high in almost all plant species, and it was confirmed that the presence and absence of the pro-domain in the N-terminus extension were clearly divided into type I and type II metacaspases on the phylogenetic tree (Figure 2). The metacaspases are also classified into type I and type II metacaspases on the basis of the linker region between p20 and p10 domains [18,47].

OsMC8 has a very short N-terminus extension, and it is not clear whether it actually functions as a pro-domain, but it is, nonetheless, included in type I (Figure 2). Similarly, primitive organisms such as eubacteria and ancestral eukaryotes also have type I metacaspases, which contain either a very short N-terminal region or lack one entirely [48]. For example, *Aureococcus anophagefferens* (AaMC1) and *Guillardia theta* (GtMC1) lack the N-terminal region completely [48]. Thus, the N-terminal domain may appear as a derived trait absent in the early evolutionary type I metacaspases of eukaryotes. It can be found that the clade having the zinc-finger motif is also clearly divided into two within the type I group (Figure 2). This might predict that pro-domain cleavage and the presence of the zinc-finger motif may be important in the function of metacaspases. All metacaspases clearly differentiate between subunits p20 and p10, and amino acid residues that play a role in the active dyad. The putative calcium binding sites are well conserved in p20, suggesting that the activity of type I and type II metacaspases is regulated by calcium ions (Figure 2). Almost all metacaspases have similar domain configurations or no additional domains. However, cucumber Csa3G129470 exhibits the longest C-terminus extension that occurs, and an additional peptidase domain is predicted (Figure 2). This may be a false prediction, as two closely replicated metacaspases in the assembly might be linked. This is because this form does not appear in most plant species. If not, it may be one of the evolutions of a more complex functional plant metacaspase. As genome sequencing in various plants progresses, metacaspases of more diverse genome configurations can be identified, and research on their functions is expected.

## 4. Metacaspase Functions in Development, Biotic, and Abiotic Stresses

Metacaspases are known to play an important role in developmentally regulated PCD in plants [18,47]. The expression specificity of metacaspases in different tissues and organs has been reported in various plants [46,50,51,52]. In grape, type II metacaspase *VvMC6* is expressed in floral tissue and exhibits low expression patterns in other tissues. Function of VvMC6 might be flower formation and ovule development in grapes. Transcription levels of type I *VvMC1*, *VvMC3*, and *VvMC4* increases during endosperm abortion in seedless grape. Thus, grape metacaspase function is involved in developmental regulation in various tissues. The function of type II AtMC4 and AtMC9 is involved in leaf senescence, because gene expression of these genes is highly up-regulated in senescence conditions [53]. A putative functional homologue of animal Bax in *Arabidopsis* is cell growth defect factor 1 (Cdf1), which promotes proapoptotic Bax-like cell death via enhancing reactive oxygen species (ROS) [54]. Overexpression of *Arabidopsis Cdf1*-*related gene Responsive to Senescence* (*CRS*) increases gene expression of *AtMC4* and *AtMC9* during senescence [53]. It would be possible to predict that metacaspases may be involved in development-related programmed cell death. However, there are still few known metacaspase transgenic plants that are affected by distinct developmental stages. This is probably due to the redundancy of metacaspase functions that are duplicated in plants. For example, petunia type I metacaspase *PhMC1*-RNAi transgenic plants exhibit normal growth phenotypes, but flower senescence is enhanced [47]. It is necessary to investigate a more certain phenotype through multiple knockout or gene silencing of metacaspase genes in the same clade.

**Figure 3:**
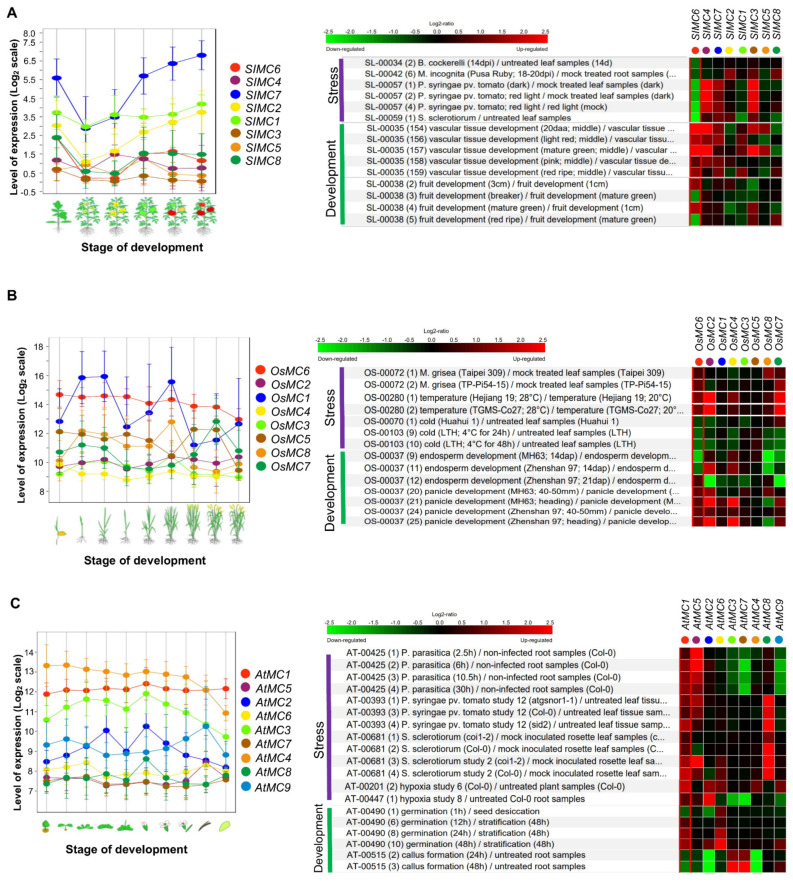
Gene expression analysis of *metacaspase* using the Genevestigator tool. (**A**–**C**) Expression data of tomato, rice, and Arabidopsis metacaspase transcripts were retrieved from Genevestigator at developmental stages and in response to different biotic and abiotic stress conditions [60]. Transcript abundance in microarray datasets of developmental stages was retrieved for eight tomato *SIMCs*, eight rice *OsMCs*, and nine Arabidopsis *AtMCs*, and analyzed. In perturbations, fold change in expression as compared to respective untreated/control sample was retrieved for each biotic and abiotic stress conditions. Up-regulation and down-regulation are shown by red and green colors, respectively.

Based on the Genevestigator tool, gene expressions of rice, tomato, and Arabidopsis metacaspase in developmental stages were analyzed (Figure 3). Various expression patterns in development were shown. Although not all metacaspases show the same expression patterns in the three plant species, type II *SIMC7* and type I *SIMC2* exhibit a precise pattern which increased according to the maturation process of tomatoes (Figure 3A). In particular, it can be checked that *SIMC3*, *SIMC4*, and *SIMC6* are strongly expressed in vascular tissue development (Figure 3A). It seems necessary to confirm the phenotype of tomato transgenic plants for the involvement of metacaspase in ripening. In rice, type I *OsMC1* and *OsMC8* show dynamic expression patterns in developmental stages. As a result, as the rice ripens, their expression tends to decrease significantly (Figure 3B). *OsMC2*, *OsMC7*, and *OsMC8* show reduced gene expression in endosperm development. *OsMC2* and *OsMC4* exhibit strong gene expression in panicle development (Figure 3B). Although it can be predicted that the function of OsMC in rice plays an important role in development, the phenotype using rice transgenic plant has not yet been confirmed. It has been reported that type II OsMC4 is involved in coping with environmental stress [55]. *OsMC4* overexpression in rice calli shows enhanced ER stress and salinity stress tolerances [55]. In *Arabidopsis*, type I *AtMC2* and *AtMC3* increase until the vegetative stage (Figure 3C), but there are no clear developmental phenotypes yet. *AtMC2* knockout mutants exhibit increased levels of cell death upon treatment with the plant-defense activator benzothiadiazole [31].

As previously reported, AtMC2 appears to have a role in hypersensitive cell death. In the case of type I *AtMC1*, there is no significant change in gene expression in the whole developmental process (Figure 3C). The single mutant phenotype of atmc1 looks smaller than the wild-type Col-0 plant [39,56]. However, the authors did not mention that single mutants *atmc1*, *atmc2*, and the double mutant *atmc1atmc2*, do not display any visible abnormal phenotypes under normal growth conditions [56,57]. Although there is a difference in the growth of type I metacaspase mutants, it appears that AtMC1, AtMC2, and AtMC3 function could be associated with lesion simulating disease resistance 1 (LSD1)-associated runaway cell death regulation [57]. *AtMC1* knockout plants exhibit delayed cell death upon *P. syringae* pv. *tomato* (*avrRpt2*) infection and more resistance to both *P. syringae* (empty vector control) and *P. syringae* (*avrRpt2*) than Col-0 WT plants [56]. AtMC1-mediated innate immunity might appear in a plant age-dependent manner because the results are different depending on the plant stage. Interestingly, AtMC1 can modulate pre-mRNA splicing, including Sm-like4 (LSM4) [39,56,57]. AtMC1 seems to have quite a variety of features. This is because there has been no report so far that caspase directly regulates genes involved in pre-mRNA splicing. Thus, type I AtMC1, 2 and 3 may play important roles in the regulation of plant apoptosis and resistance to pathogen infection.

In the Genevestigator analysis, *AtMC8* shows highly enhanced gene expression upon *P. syringae* and *Sclerotinia sclerotiorum* (cottony soft rot). *AtMC7* and *AtMC9* exhibit inhibited gene expression upon *Phytophthora parasitica* (soilborne pathogen). Gene expression of *AtMC1* and *AtMC5* is upregulated upon *P. syringae*, *P. parasitica*, and *S. sclerotiorum* (Figure 3C). Gene expression for these pathogens is similar in tomato metacaspase. *SIMC4*, *SIMC3*, and *SIMC7* exhibit enhanced gene expression upon *P. syringae* but *SIMC6* is highly reduced by *P. syringae*, *S. sclerotiorum*, *Meloidogyne incognita* (root-knot nematode), and *Bactericera cockerelli* (tomato/potato psyllid) infection (Figure 3A). In most rice *OsMCs*, gene expression patterns did not change significantly in compatible and incompatible interactions upon *Xanthomonas oryzae* pv. *Oryzae* [22]. The gene expression levels of *OsMC1* and *OsMC7* were significantly reduced by compatible interaction. On the other hand, *OsMC2* and *OsMC8* are strongly induced by incompatible interaction [38]. Gene expressions of *OsMC6*, *OsMC7*, and *OsMC8* are slightly upregulated by *Magnaporthe grisea* infection, but, similarly, most *OsMCs* do not respond to rice pathogen (Figure 3B). Interestingly, mRNA levels of *OsMC2*, *OsMC6*, and *OsMC7* were induced by temperature stress (Figure 3B). In cold stress conditions, gene expression of most *OsMCs* exhibits reduced patterns (Figure 3B). It is implied that the function of metacaspase coexists with a positive role in plant immunity against pathogen invasion and a negative role in pathogen-inhibited responses. Metacaspases, such as mammalian caspases, are cysteine proteases involved in programmed cell death and plant immunity in plants. Thus, metacaspases can participate in various ways in developmental programmed cell death and defense-related cell death. These functions are believed to depend on metacaspase substrate proteins.

Metacaspases can be major targets of pathogen effector proteins. For example, *Salmonella* type III secreted effector protein SifA, which contains a functionally active caspase-3 cleavage site which can utilize caspase-3 to invade and persist during infection [58]. *Shigella flexneri* evaded pyroptosis mediated by caspase-11 or caspase-4 using the effector OspC3 protein which can assist in arginine ADP-riboxanation of caspase-11/-4 and the blocked autoprocessing of caspases [59]. If metacaspases and pathogen interactions in plant immunity evolve like an arms-race, they are likely to be targets of pathogen effectors or manipulations such as caspases. Although there are no reports of protein interactions between plant metacaspases and pathogen effectors, they may well be possible, as shown by the results of mammalian caspases.

## 5. Emerging Physical Interactions between Metacaspase and Autophagy

Caspase has been identified to directly interact with key autophagy-related (Atg) proteins [61,62]. For example, the BAD-BAX-caspase-3 cascade is known as a canonical apoptosis pathway, and it has been shown that this cascade can control the pool of restrictive synaptic vesicles by modulating autophagy [63]. In the hippocampus of *Casp-3* KO mice, Atg3, Atg4, Atg7, Atg9, and Beclin-1 protein accumulations are enhanced, as well as autophagosome-like structures [63]. Previous research results have found that several ATG proteins can be cleaved by caspase-3 in vitro [64]. These results suggest that caspase directly targets autophagy proteins and regulates protein levels. Thus, an increase in autophagy-related protein in *Casp-3* KO mice can result from diminished proteolysis by caspase-3. Nuclear protein TP53INP2 can shuttle from the nucleus to cytosol, and acts as a positive regulator of autophagy when in a nutrient depletion condition [65]. The TP53INP2 interacts with the LC3-interacting region (LIR) motif of Atg8 proteins [66]. Recently, it was found that TP53INP2 controls death receptor-induced apoptosis and interacts with caspase-8 to regulate its ubiquitination levels [67]. Physical interaction between pro-caspase-8 and autophagy proteins has been found that death effector domains (DEDs) in the pro-caspase-8 can associate with ATG5-ATG12-FADD and then pro-caspase-8 forms a stressosome complex upon cell stress [68]. In earlier studies, Beclin-1 was known as a substrate for caspase-3 and cleavage of Beclin-1 by caspase-3 can contribute to inactivating autophagy leading to increased apoptosis [69,70]. Thus, caspase can regulate autophagy-related proteins through caspase activity. 

**Figure 4:**
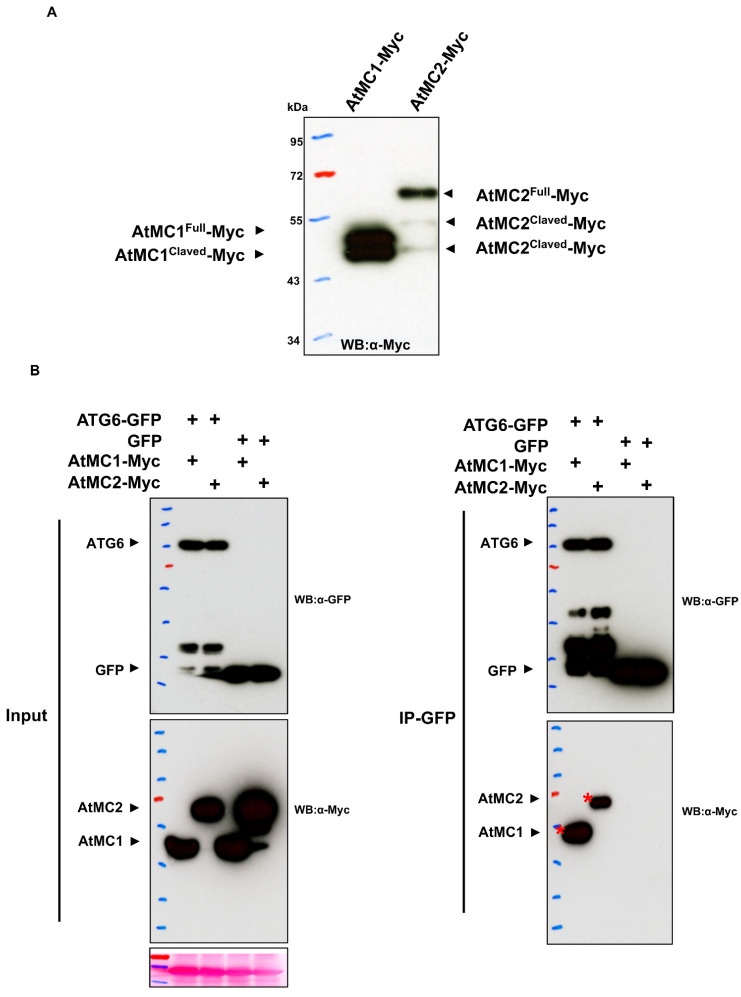
Arabidopsis type I metacaspases associate with autophagy ATG6 protein. (**A**). Auto-processing of AtMC1 and AtMC2 *in planta*. Transient overexpression of *AtMC1-Myc* and *AtMC2-Myc* results in auto-processing in *Nicotiana. benthamiana*. *AtMC1-Myc* or *AtMC2-Myc* were transiently expressed in *N. benthamiana* leaves. At 2 dpi, samples were harvested, and then Western blot analysis was performed with an anti-Myc antibody. (**B**). Coimmunoprecipitation (CoIP) shows that metacaspases associate with autophagy in the ATG6 protein. Transient co-expression assays of *ATG6-GFP* or *GFP* control with *AtMC1-Myc* or *AtMC2-Myc* were performed in *N. benthamiana* leaves. Immunoblots show the presence of proteins in total extracts (input) and after immunoprecipitation with anti-GFP beads (IP-GFP). Red asterisks represent full-length proteins.

In plants, it has already been reported that their functions genetically interact between autophagy and metacaspase mutants [39,71]. The *atmc1 atg5* or *atmc1 atg18* double mutants exhibit an additive effect on HR cell death suppression and negatively regulate senescence [39]. However, nothing is known about regulatory action through protein interaction yet. As previously reported, it is believed that AtMC1 and AtMC2 have the characteristic of self-cleavage when expressed in plants, and that their function is activated similarly to caspase (Figure 4A). From the viewpoint of the animal system [69], coIP was performed to confirm the possibility that plant autophagy and metacaspases regulate each other through protein-protein interactions. Based on coIP experiments, *Arabidopsis* ATG6-GFP associates with AtMC1-Myc and AtMC2-Myc proteins (Figure 4B). Interestingly, the full proteins of metacaspase AtMC1 and AtMC2 interact with ATG6. Plant ATG6 is the ortholog of yeast Vps30/Atg6, and mammalian BECN1/Beclin-1 [72]. Perhaps, even in a form that is not an autoclaved metacaspase, it may be able to perform some function through interaction with autophagy proteins. Future studies are expected on how the interaction between metacaspases and autophagy-related proteins regulates apoptosis and aging functions. There is a report that *Petunia* × *hybrida* autophagy *PhATG6* silencing affects metacaspase 1 (*PhMC1*) gene expression [73]. Conversely, caspase has been identified as a regulator of autophagy-related genes in chondrocytes as part of physiological cartilage development [74]. This suggests that autophagy regulates the gene level of metacaspases in the aging process, and this is obviously a process that occurs in autophagy and unknown transcriptional regulators.

## 6. Conclusions

From an evolutionary point of view, the prediction of the function of metacaspase, a conformational analogue of caspase, will be somewhat similar. However, the distinction between functions such as initiator and effector of caspases is not yet clear. Regarding mutant phenotypes, metacaspase is also involved, such as in immunity and developmental PCD, in which caspase is involved. Further studies suggest that the function of metacaspases may be in the regulation of autophagy-related apoptosis in genetical and physical interactions. Knowledge of whether the functions of type I and type II are differentiated, and various approaches, are required to determine target proteins.
